# Granulomatosis With Polyangiitis in Untreated Graves’ Disease: A Rare Event

**DOI:** 10.7759/cureus.29742

**Published:** 2022-09-29

**Authors:** Syed Ahmad Moosa, Christian M Mendez, Zuleyka Sanchez, Luis Mariano Cabrera, Sameera Ishtiaq

**Affiliations:** 1 Internal Medicine, St. John's Episcopal Hospital, New York, USA; 2 Research, Bangladesh Medical Association of North America, New York, USA; 3 Internal Medicine, St John's Episcopal Hospital, New York, USA

**Keywords:** rituximab therapy, thyroid-storm, anca associated vasculitis, coexistence, graves´disease, granulomatosis with polyangiitis (gpa)

## Abstract

Antithyroid medications can cause antineutrophil cytoplasmic antibody (ANCA) vasculitis. However, no literature in English describes the coexistence of granulomatosis with polyangiitis (GPA) and untreated Graves’ disease. We present a 19-year-old female with thyroid storm and additional complaints of cough, hemoptysis, nasal discharges, polyarthralgia, and skin lesions. Imaging showed peri-hilar cavities and acute-on-chronic sinusitis. Elevated cytoplasmic pattern antineutrophil cytoplasmic antibody (C-ANCA) and anti-proteinase-3 antibody levels plus histopathology of the nasal and skin biopsies suggested GPA. Propranolol, methimazole, and potassium-iodide resolved the thyroid storm. Induction therapy (steroids, rituximab) for GPA provided relief of chronic symptoms stressing the importance of early recognition and swift initiation of treatment

## Introduction

Graves’ disease, an autoimmune disorder, is the most common cause of hyperthyroidism [1]. Granulomatosis with polyangiitis (GPA), formerly called Wegener's granulomatosis, is an uncommon immune-mediated disease of unknown etiology included in the group of anti-neutrophil cytoplasmic antibody (ANCA)-associated small vessel vasculitides. Antithyroid medications can cause ANCA vasculitis [2-6]. But our extensive review of existing literature in English could not identify the coexistence of GPA and Graves’ disease in patients who have not received any anti-thyroid medications. In this article, we describe and discuss a rare case of a young adult female with such rare occurrence.

## Case presentation

A 19-year-old female student with hyperthyroidism was evaluated in the emergency department (ED) of the hospital for thyrotoxicosis. The patient was in her usual state of health until approximately one year before presentation when she developed a chronic productive cough, hemoptysis, bloody nasal discharges, weight loss, and pain in multiple joints. These symptoms were accompanied by painful skin lesions that occasionally bled. These lesions appeared first on the face and gradually involved the back, arm, and axilla. Two months before the presentation to the ER, she developed intermittent fever, diarrhea, palpitations, sweating, and dizziness that suddenly worsened a day before this visit. She had no chest pain, abdominal pain, urinary symptoms, or menstrual cycle changes.

She was diagnosed with hyperthyroidism in another hospital earlier in the month, and without a specific cause of hyperthyroidism being established, she was commenced on oral propranolol (20 mg once daily). Two days before her presentation at the ED, she was seen at an endocrinology clinic. The dosage of propranolol was adjusted to 20 mg twice daily; however, a further work-up was rescheduled to a later date at the patient’s request. On evaluation, she reported no tremors or gastrointestinal problems. The patient was born in Guatemala and made her most recent visit there a year ago. There was no family history of similar symptoms or chronic illness. She had no surgical history or known allergies, and her immunization was up to date. Her only medication was propranolol, and she has no history of using illicit substances.

She had a temperature of 101.5°F, blood pressure of 140/85 mm Hg, a pulse of 140 beats per minute, respiratory rate of 30 breaths per minute, and oxygen saturation of 94% on ambient air. She appeared to be distressed, uncomfortable, diaphoretic, and mildly anemic. Skin assessment revealed punched-out ulcerations with minimal bloody drainage located on the right side of the face, back, left arm, right arm, and left axilla. Other aspects of the physical examination including the musculoskeletal system were normal. Laboratory and microbiological test results are shown in Table 1. The results of the rheumatological studies and some microbiological studies (related to *Histoplasma*, and *Aspergillus*) were not available until the end of week two as the tests were processed by a third-party lab. 

**Table 1:** Laboratory and microbiological data ANA: Antinuclear antibody, C-ANCA: Cytoplasmic pattern antineutrophil cytoplasmic antibodies, ANCA: Antineutrophil cytoplasmic antibodies, PR3: Proteinase-3, MPO: Myeloperoxidase antibody, GBM: Glomerular basement membrane, OH: Hydroxy, PAS: Periodic acid–Schiff, GMS: Grocott methenamine silver, DNA: Deoxyribonucleic acid, PCR: Polymerase chain reaction, IgE: Immunoglobulin E, IgG: Immunoglobulin G, HIV: Human immunodeficiency virus, Hepatitis Bs antigen: Hepatitis B surface antigen, RSV: Respiratory syncytial virus, SARS-COV-2: Severe acute respiratory syndrome coronavirus 2, RNA: Ribonucleic acid, RT-PCR: Reverse transcription polymerase chain reaction, T3: Triiodothyronine, T4: Thyroxine, p-ANCA: Perinuclear anti-neutrophil cytoplasmic antibodies

Variable	Reference Range		At Presentation
White cell count (/mm3)	4500-13000		9600
Hemoglobin (gm/dL)	12.0-16.0		10.3
Hematocrit (%)	36.0-46.0		31.3
Erythrocyte sedimentation rate (mm/h)	0-20		104
C-reactive protein (mg/dL)	< 10		5.7
Procalcitonin (ng/mL)	< 0.1		0.09
Thyroid Profile			
Thyroid-stimulating hormone (mIU/L)	0.47-4.68		< 0.02
Free T3 (ng/dL)	0.78-2.19		2.64
Free T4 (pg/mL)	1.80-4.60		7.97
Thyroid Antibodies			
Thyroid peroxidase antibodies (IU/mL)	< 34.9		110
Thyroglobulin antibodies (IU/mL)	< 20		< 20
Thyroid-stimulating hormone receptor antibodies (IU/L)	0.0-0.55		2.60
Iron Studies			
Serum iron (μg/dL)	37-170		28
Total iron-binding capacity (μg/dL)	265-497		198
Transferrin level (mg/dL)	206-381		156
Ferritin level (ng/dL)	6.24-137		209
Rheumatologic Studies			
Rheumatoid factor (IU/mL)	0-13		28
Cyclic-citrullinated peptide (Units)	≤ 19		< 8
ANA titer	Negative		Negative
c-ANCA screen	Negative		Positive
c-ANCA titer	≤ 1:20		1:80
p-ANCA	Negative		Negative
Anti-PR3 antibody (units)	≤ 20		159.8
Anti-MPO antibody	Negative		Negative
Atypical ANCA (x-ANCA)	Negative		Negative
Anti-GBM antibody (units)	0-20		4
Serum angiotensin-converting enzyme (units/L)	14-82		34
Vitamin D 1,25 OH (pg/mL)	19.9-79.3		12.8
Microbiological and Related Studies			
QuantiFERON-TB Gold Plus (QFT-Plus)	Negative		Negative
Sputum smears for acid-fast bacilli (fluorochrome stain)	Three negative smears		Three negative smears
Sputum smear for Gram stain	Negative		Negative
Sputum fungal smear (PAS and GMS stain)	Negative		Negative
Blood culture	Negative		Negative
Urine culture	Negative		Negative
Sputum culture for acid-fast bacilli	Negative		Negative
Sputum culture for bacteria	Negative		Negative
Sputum culture for fungus	Negative		Negative
Urine for Chlamydia DNA PCR	Not detected		Not detected
Urine for Gonorrhea DNA PCR	Not detected		Not detected
Serum *Histoplasma* mycelial antibody titer (complement fixation)	<1:8		<1:8
Serum *Histoplasma* antibody with yeast titer	<1:8		<1:8
Urine for *Histoplasma* antigen enzyme immunoassay (ng/mL)	<0.2		0
Serum for *Aspergillus flavus* allergen IgE	Negative (<1:1)		Negative
Serum for Aspergillus fumigatus allergen IgG	Negative (<1:1)		Negative
HIV 1&2 antigen/antibody, 4^th^ generation	Negative		Negative
Hepatitis Bs antigen	Negative		Negative
Hepatitis Bs antibody (mIU/mL)	> 1000	Non-reactive/non-immune: <5 Indeterminate: 5-12 Reactive/Immune: >12	
Hepatitis B Core antibody	Negative		Negative
Hepatitis C antibody	Negative		Negative
Influenza A antigen	Negative		Negative
Influenza B antigen	Negative		Negative
RSV antigen	Negative		Negative
SARS-COV-2 RNA (RT-PCR)	Negative		Negative

Urinalysis on admission revealed numerous red blood cells. An electrocardiogram showed sinus tachycardia. The Burch-Wartofsky point scale (BWPS) that helps predict the probability of thyrotoxicosis independent of thyroid hormones showed a score of 45 (Table 2), suggesting a thyroid storm [7]. She was initially managed with intravenous fluids as well as oral propranolol, oral potassium iodide, and oral methimazole in the ED, followed by admission to the internal medicine telemetry floor. 

**Table 2:** Burch-Wartofsky point scale (BWPS) scoring

Factor	BWPS
Thermoregulatory dysfunction: Temperature of 101.1 °F	10
Central nervous system effects: Absent	0
Gastrointestinal-hepatic dysfunction: Moderate diarrhea	10
Heart rate: ≥ 140 bpm	25
Congestive heart failure: Absent	0
Atrial fibrillation: Absent	0
Precipitating event: None	0
Total score	45

Thyroid ultrasound showed mildly hyper-vascular thyroid with no focal thyroid nodule, suggestive of thyroiditis. The radioactive iodine uptake (RAIU) test could not be performed because the patient was administered multiple doses of potassium iodide during admission. In the hospital, symptoms of thyrotoxicosis resolved. The endocrinologist re-evaluated the patient and recommended the continuation of oral propranolol and methimazole and the discontinuation of potassium iodide.

**Figure 1:**
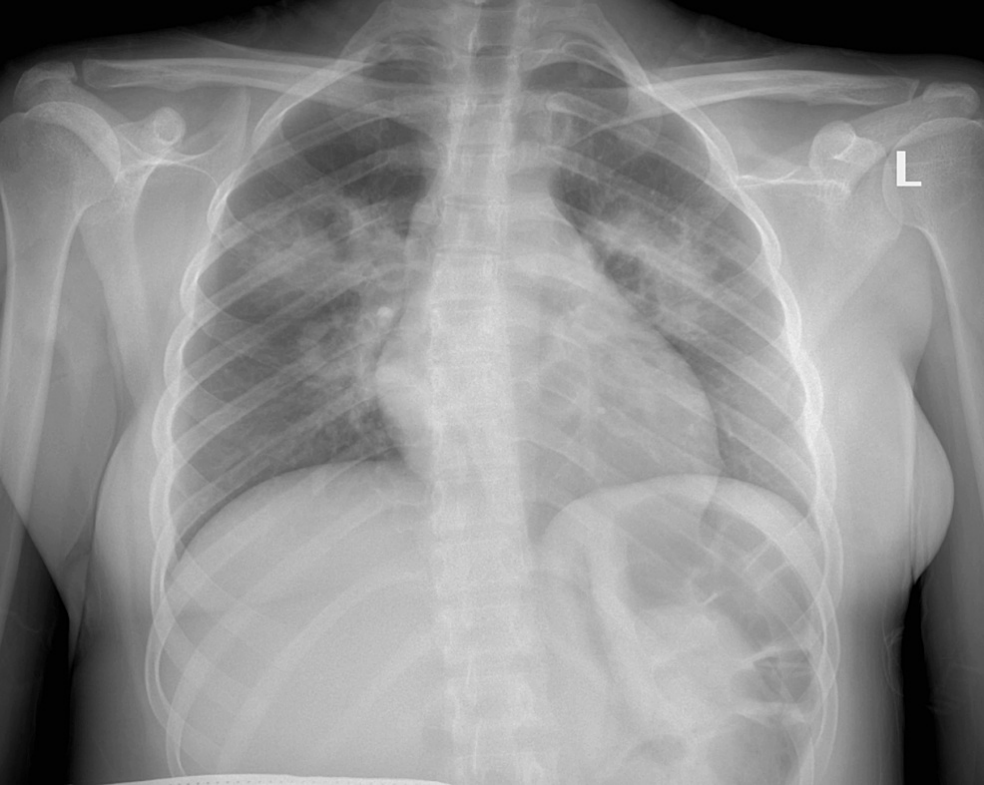
A chest X-ray shows bilateral perihilar opacities without lymphadenopathy

**Figure 2:**
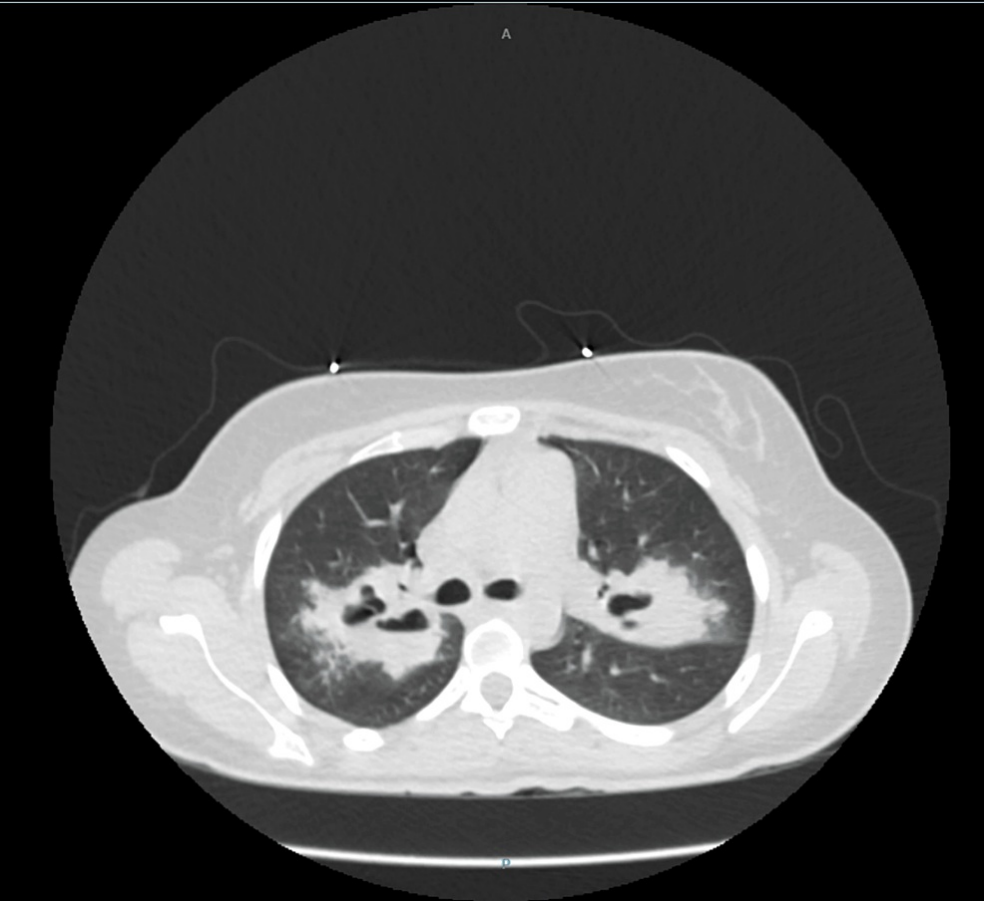
The CT images of the chest (one slice shown above) without contrast show multiple cavities with thick and irregular walls with adjacent lung consolidation. No lymphadenopathy.

**Figure 3:**
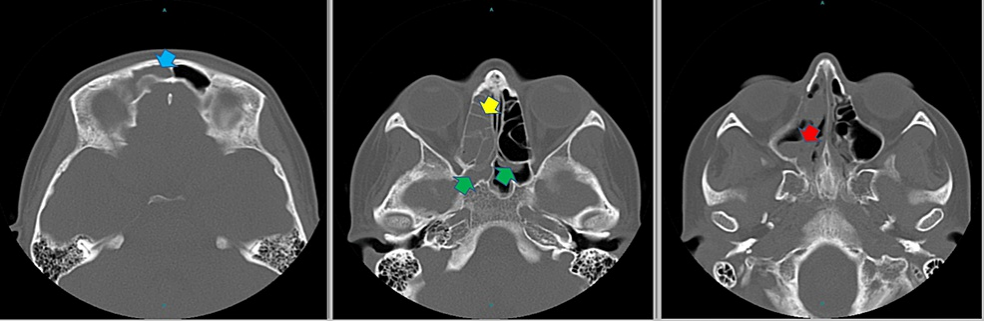
The CT images of the paranasal sinuses show right frontal (blue arrow), right ethmoid (yellow arrow), right maxillary (red arrow), and bilateral sphenoid acute-on-chronic sinusitis (green arrows).

A single-view anteroposterior radiograph of the chest revealed bilateral hilar opacities (Figure 1), and the chest computed tomography (CT) showed thickened wall peri-hilar cavities with adjacent lung consolidation (Figure 2). Computed tomography of the nasal sinuses revealed acute-on-chronic right frontal, ethmoid, maxillary, and bilateral sphenoid sinusitis (Figure 3). A transthoracic echocardiogram did not reveal any vegetation. The microscopic hematuria resolved spontaneously.

**Figure 4:**
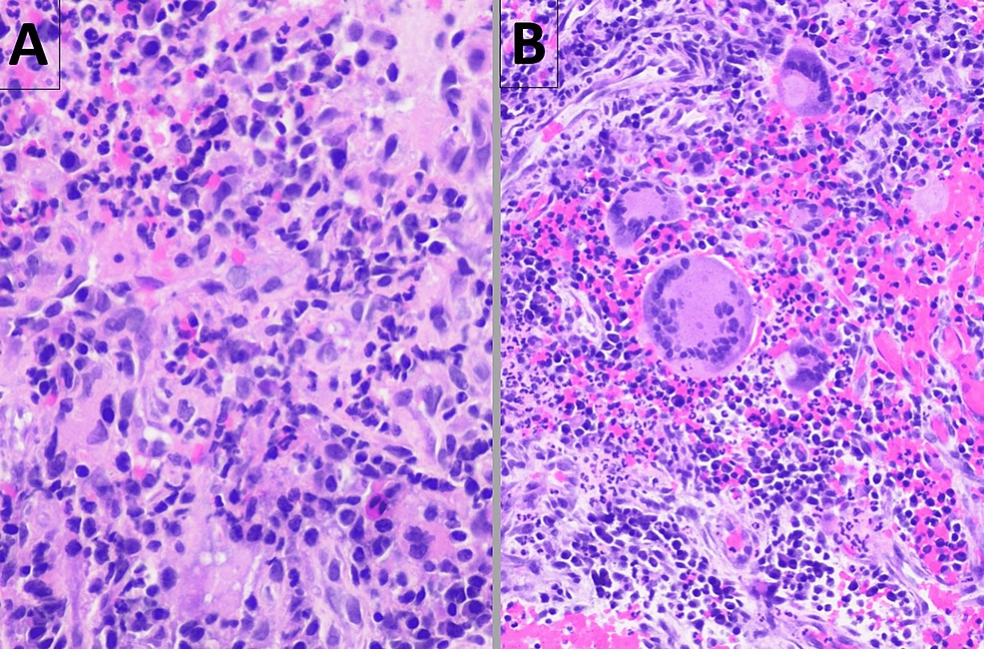
Histopathology of the left nasal lateral wall biopsy specimen shows poorly defined granulomas with an admixture of acute and chronic inflammatory cells that include neutrophils, plasma cells, lymphocytes, histiocytes (Panel A), and a few eosinophils with multinucleated giant cells (Panel B). The histopathologist noted that no defined foreign body was identified, and examination with polarized light did not demonstrate birefringence.

**Figure 5:**
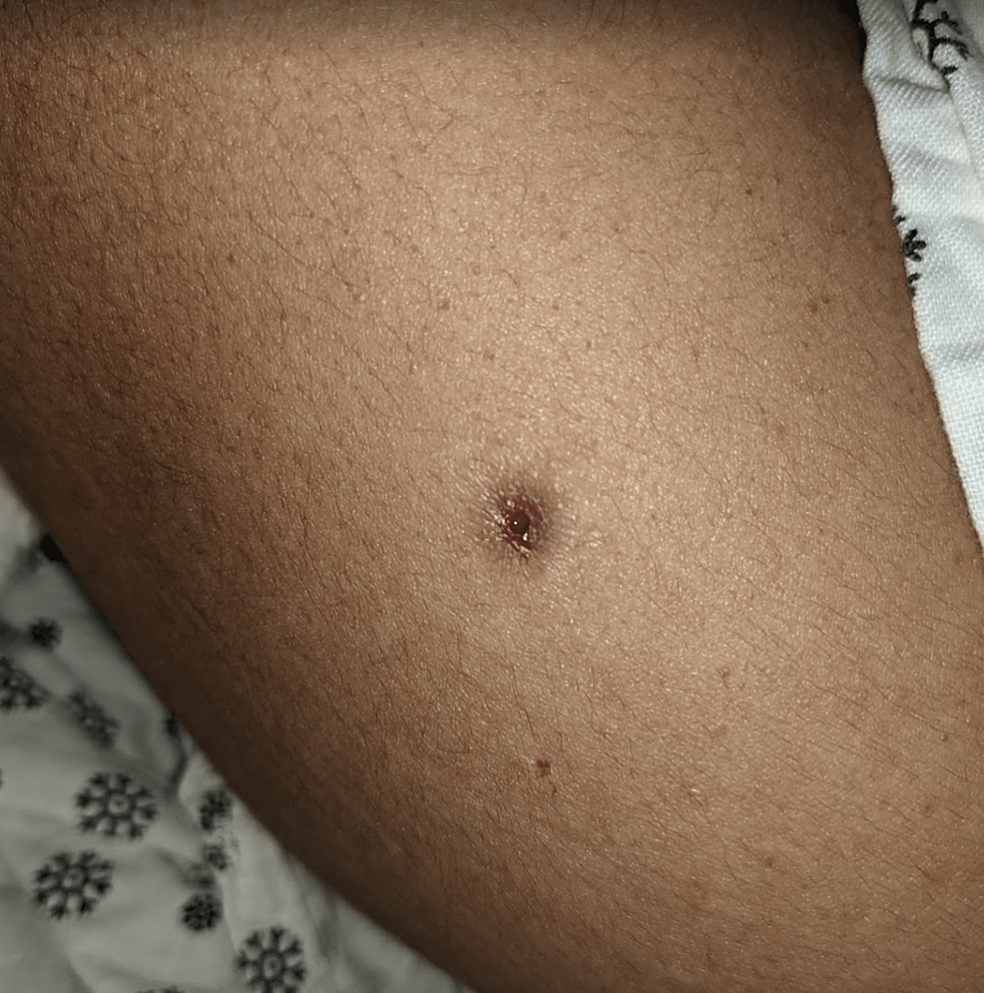
Punched-out ulceration (approximately 3 mm in diameter) with minimal bloody drainage located on the right arm. Similar lesions were seen on the right side of the face, back, left arm, and left axilla.

**Figure 6:**
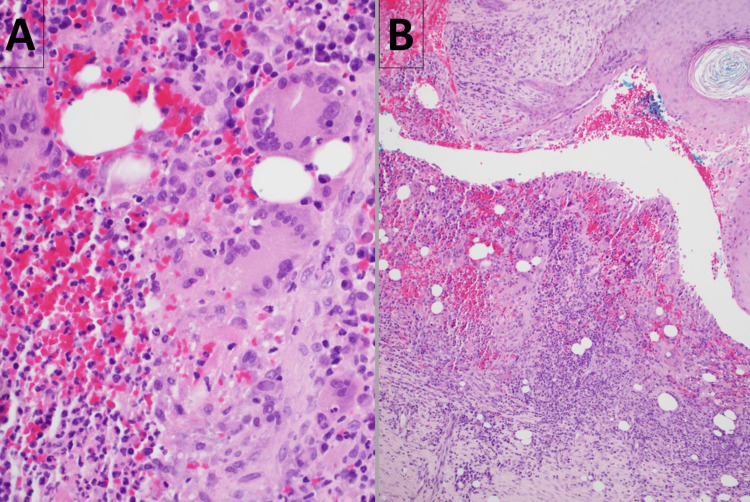
Histopathology of punch biopsy of one of the ulcerated skin lesions on the upper back shows dermal infiltration of histiocytes, some multinucleated (Panel A), as well as lymphocytes, and plasma cells. There are extravasated erythrocytes and fibrin adjacent to blood vessels (Panel B). In the context of cavitary lung lesions, elevated C-ANCA and anti-PR3 antibodies, the histopathologists’ opinion was that the microscopic findings were consistent with granulomatosis with polyangiitis. C-ANCA: Cytoplasmic pattern antineutrophil cytoplasmic antibodies, PR3: Proteinase-3

The histopathology of the biopsy specimen from the left lateral nasal wall was compatible with the diagnosis of GPA, revealing multiple fragments of granulation tissue, with an admixture of acute and chronic inflammatory cells that included neutrophils, plasma cells, lymphocytes, histiocytes, and a few eosinophils with multinucleated giant cells containing cytoplasmic clearing; however, there was no evidence of vasculitis (Figure 4). The histopathological findings of the punch biopsy specimen from one of the ulcerated skin lesions (Figure 5) were not entirely specific, revealing granulomatous dermatitis with extravasated erythrocytes, associated fibrin, and some suppurative inflammation (Figure 6).

## Discussion

Differential diagnosis

Our patient presented with symptoms of thyrotoxicosis (such as intermittent fever, diarrhea, palpitations, sweating, and dizziness) accompanied by chronic productive cough, hemoptysis, thick bloody nasal discharges, multiple joint pains, and painful skin lesions with bloody discharge for one year. The differential diagnosis of this patient includes Graves’ disease, Hashitoxicosis, toxic adenoma, toxic multinodular goiter, pulmonary tuberculosis, GPA, microscopic polyangiitis (MPA), and eosinophilic granulomatosis with polyangiitis (EGPA). Several published case reports highlighted the co-occurrence between ANCA vasculitis and hyperthyroidism due to antithyroid medications. However, none of them emphasized the co-existence of GPA and Graves’ disease [2-6].

Graves' Disease

Graves’ disease, an autoimmune disorder, is caused by thyroid-stimulating immunoglobulins (TSIs) that stimulate thyroid gland growth and thyroid hormone synthesis [8]. The clinical manifestations of Graves' disease are symptoms and signs resulting from hyperthyroidism and may also include diffuse goiter and ophthalmopathy. The presence of palpitations, diarrhea, sweating, dizziness, and intermittent fever suggests Graves’ disease. In addition, the age of the patient, and findings of a thyroid antibody panel and thyroid profile are consistent with the diagnosis of Graves' disease (as seen above in Table 2).

Hashitoxicosis

Hashitoxicosis refers to the hyperthyroid phase of Hashimoto’s thyroiditis and is used to describe patients who initially present with hyperthyroidism and high radioiodine uptake, followed by autoimmune thyroid destruction and lymphocytic infiltration [9]. Hashitoxicosis has features similar to Graves' disease except for low radioiodine uptake by the thyroid gland and negative TSI [10]. This patient’s symptoms were consistent with Graves’ disease and Hashitoxicosis. However, based on the TSIs, this patient was unlikely to have hyperthyroidism due to Hashitoxicosis.

Toxic Adenoma and Toxic Multinodular Goiter

Toxic adenoma and toxic multinodular goiter are also causes of hyperthyroidism [11]. Our patient showed manifestations of hyperthyroidism. However, there was no evidence of thyroid nodules on thyroid ultrasound, thus excluding toxic adenoma and toxic multinodular goiter.

Pulmonary Tuberculosis (TB)

Pulmonary TB, caused by *Mycobacterium tuberculosis*, is suspected in patients with relevant clinical manifestations (cough of > 2 to 3 weeks duration, lymphadenopathy, fever, night sweats, and weight loss) and epidemiologic factors (history of prior TB infection, known or possible TB exposure, and past or present residence in or travel to an area where TB is endemic [12]. In our patient, pulmonary TB was initially favored based on the findings of CT of the chest, chronic productive cough, hemoptysis, weight loss, and a visit to an endemic region (Guatemala). However, negative sputum culture and QuantiFERON-TB Gold Plus ruled out the diagnosis.

Granulomatosis With Polyangiitis

Granulomatosis with polyangiitis, formerly called Wegener's granulomatosis, is an uncommon immune-mediated disease of unknown etiology included in the group of ANCA-associated small vessel vasculitides. It is characterized by necrotizing vasculitis of small-to-medium size vessels (capillaries, venules, arterioles, arteries, and veins). It produces granulomatous inflammation of the kidneys and upper and lower respiratory tract [13-14]. More than 80% of GPA patients are positive for ANCA. The disease is diagnosed based on granulomas in the upper airways, necrotizing vasculitis, and glomerulonephritis [15]. The presence of chronic productive cough with hemoptysis, thick bloody nasal discharges, multiple joint pains, and painful skin lesions in this patient with elevated c-ANCA levels and anti-proteinase-3 (PR3) antibody strongly suggests the diagnosis of GPA. In addition, the nasal wall and skin biopsy results supported our diagnosis of GPA.

Microscopic Polyangiitis 

Microscopic polyangiitis most commonly manifests as perinuclear anti-neutrophil cytoplasmic antibodies (p-ANCA) positive necrotizing glomerulonephritis or pulmonary capillaritis and presents with non-specific symptoms [16]. The presence of upper airway involvement and positive anti-PR3 antibody in this patient makes the diagnosis of MPA unlikely.

Eosinophilic Granulomatosis With Polyangiitis 

Eosinophilic granulomatosis with polyangiitis, previously called Churg-Strauss syndrome (CSS), is usually a p-ANCA positive vasculitis characterized by allergic rhinitis, asthma, and peripheral blood eosinophilia [17]. It was excluded based on the absence of asthma, allergies, nasal polyps, and eosinophilia.

Diganostic testing

The diagnostic procedures included imaging, biopsy, and laboratory tests. Based on the clinical suspicion, we diagnosed hyperthyroidism on the basis of low thyroid stimulating hormone (TSH) and elevated triiodothyronine (T3) and free thyroxine (fT4) levels. The next step was to confirm the cause of hyperthyroidism. Thus, Graves' disease was diagnosed by elevated thyroid stimulating immunoglobulin (TSI) levels (as seen above in Table 1). The TSI is a thyroid-stimulating hormone receptor antibody (TRAb). Studies have shown that the presence of a TRAb has a sensitivity and specificity of 97% and 99% for diagnosing Graves' disease [18]. A thyroid ultrasound excluded the presence of hot or cold thyroid nodules. Radioactive iodine uptake and scan (RAIU+S), not performed on this patient, is also used to confirm the diagnosis of Graves’ disease [19].

The clinical (chronic productive cough with hemoptysis, thick bloody nasal discharges, multiple joint pains, keratitis, and painful skin lesions) and laboratory (such as elevated c-ANCA levels, raised anti-PR3 antibody, and positive rheumatoid factor) manifestations suggested the diagnosis of GPA. Depending on the disease severity, positive ANCA is seen in approximately 82% to 94% of patients with GPA and MPA, while anti-PR3 antibody is associated with 65% to 95% of patients with GPA only [20-21]. Additional laboratory testing included serum creatinine, urinalysis with urine sediment, erythrocyte sedimentation rate, C-reactive protein, and a complete blood count. A chest radiograph and CT scan were performed to detect pulmonary involvement in our patient due to the suspicion of GPA. Biopsy of lungs, kidneys, skin or nasal wall should be performed before starting long-term treatment [22]. In our patient, histopathology of the biopsy specimen from the left lateral nasal wall was compatible with the diagnosis of GPA revealing multiple fragments of granulation tissue, with an admixture of acute and chronic inflammatory cells that include neutrophils, plasma cells, lymphocytes, histiocytes, and a few eosinophils with multinucleated giant cells containing cytoplasmic clearing. Furthermore, the histopathological findings of the punch biopsy specimen were not entirely specific, revealing granulomatous dermatitis with extravasated erythrocytes, associated fibrin, and some suppurative inflammation. A positive ANCA test strongly supports the diagnosis. However, tissue biopsy is the gold standard method for diagnosing GPA.

Management discussion

There are three effective treatment options for Graves’ disease: anti-thyroid drugs, radioiodine, and surgery. These treatment options are not mutually exclusive. The anti-thyroid drugs may be used for one or two years to induce remission before definitive treatment with radioiodine or surgery [23]. The results of the rheumatological studies were not immediately available. Moreover, the patient was in a state of thyroid storm which needed immediate therapy. We initially started the patient on propranolol, methimazole, and potassium iodide to control the symptoms of thyrotoxicosis (thyroid storm). The treatment regimen was readjusted to 10 mg of oral methimazole twice daily and 20 mg of oral propranolol every 8 hours with symptom improvement. When the results of positive C-ANCA and anti-PR3 antibodies were available, a decision was taken to continue with the anti-thyroid medication based on the reasons that the patient has been C-ANCA positive prior to initiating therapy and there was no clinical evidence suggesting that the patient's chronic symptoms related to the vasculitis were worsening since receiving the medications.

The goal of treatment in patients with GPA is achieved by an initial induction phase aimed at remission in patients with active disease, followed by the maintenance phase aimed at preventing relapse. The type of treatment depends upon the severity of the disease and the organ systems involved. In patients with organ- or life-threatening disease, induction is mainly achieved in three to six months by a combination of glucocorticoids with intravenous rituximab (two total doses of 1 g 14-days apart), and the maintenance of remission is achieved in 12 to 24 months with intravenous rituximab (500 to 1000 mg every six months) [24]. The rituximab for ANCA-associated vasculitis (RAVE) trial suggested an alternate rituximab induction regimen of 375 mg/m2 per week for four weeks [25]. In patients with non-organ- or non-life-threatening disease, induction is achieved by glucocorticoids combined with oral methotrexate (20 to 25 mg once weekly), and methotrexate can also be used to maintain remission. Rituximab is a reasonable alternative for those with sinusitis, rhinosinusitis, or pulmonary involvement [26]. A rituximab-based regimen was preferred over cyclophosphamide based on studies showing its comparable efficacy and less-severe side-effect profile [24]. Oral trimethoprim-sulfamethoxazole (160 mg/80 mg thrice weekly) is used as prophylaxis to prevent pneumocystis jirovecii pneumonia in all patients receiving immunosuppressive therapy.

At our institute, we initially administered pulses of methylprednisolone intravenously for three days, followed by 1 mg/kg of oral prednisone. Induction was attained by a combination of glucocorticoids with intravenous rituximab (1g every 14-days). Oral trimethoprim-sulfamethoxazole was used as prophylaxis to prevent *Pneumocystis jirovecii* infection. Topical antibiotics (moxifloxacin and erythromycin) and prednisone were used to treat the patient's keratitis. She was discharged home on oral prednisone and trimethoprim-sulfamethoxazole and was advised to follow up after 14 days with her primary care physician for the next dose of rituximab. She was also recommended additional follow-up visits to an endocrinologist, pulmonologist, and primary care physician to see her response to induction therapy and switch her to maintenance therapy.

## Conclusions

Several articles in the past have noted that antithyroid medications can cause ANCA vasculitis. However, no literature published in English, to the best of our knowledge, describes the co-existence of GPA and untreated Graves’ disease. In our brief report, we discuss a case with such a unique presentation. According to our patient’s statement, she had been suffering from chronic issues for many months and had visited multiple renowned hospitals in New York City in the hopes of finding a cure without success. Although she had been treated for hyperthyroidism, many chronic problems such as cough, hemoptysis, skin lesions, nasal discharges, etc. were never addressed.

Diagnosing and treating this young patient at our community hospital despite many logistical obstacles not only provided her with physical comfort but also brought psychological relief to her and her entire family. Based on our experience treating this patient, we recommend keeping a lookout for coexisting vasculitis in patients with another autoimmune disease, especially when some chronic symptoms cannot be explained. Our report highlights the importance of maintaining a high degree of suspicion for auto-immune vasculitis in a patient with Graves’ disease regardless of the use of antithyroid medications.
